# Initial NIST AC QHR Measurements

**DOI:** 10.6028/jres.109.028

**Published:** 2004-08-01

**Authors:** M. E. Cage, S. H. Shields, A. Jeffery

**Affiliations:** National Institute of Standards and Technology, Gaithersburg, MD 20899-8172

**Keywords:** ac quantum Hall effect, dc quantum Hall effect, frequency dependences, multifrequency bridge, quadruple-series connections, single-series connections

## Abstract

We demonstrate that dc quantized Hall resistance (dc QHR) guideline properties and dc and ac QHR values can be measured without changing sample probe lead connections at the QHR device, and report ac QHR values that converge to the dc QHR value when using four-terminal-pair ac QHR measurements. This was accomplished during one cooldown using single-series and quadruple-series connections outside the sample probe. The QHR was measured from 0 Hz to 5500 Hz in 1:1 ratio at 20 µA to ±1 part in 10^7^ uncertainties with a poor-quality QHR device. A good device would allow an order of magnitude smaller uncertainties over this frequency range. We exchanged positions of the QHR device and reference resistor in the bridge and remeasured the resistance ratios to remove dominant ac bridge effects.

## 1. Introduction

An important step in developing the ac quantized Hall resistance (ac QHR) [1–10] as an intrinsic impedance standard based on the dc QHR [11–13] is to measure the dc QHR guideline properties [14] and the dc and ac QHR values without changing sample probe lead contacts at the QHR device. (Otherwise guideline properties should be remeasured.) We show in the first ac QHR experiment at NIST that this can be accomplished in a single cooldown using multiple connections [15] to the device outside the sample probe. The device was found to not be of standards-quality; so only guideline properties needed for this particular experiment were measured (shapes of the QHR plateaus and longitudinal voltages *V_x_*, contact resistances, longitudinal resistances at the *V_x_* minima, dc QHR values for all three quantum Hall probes sets at the *V_x_* minima, and the quadruple-series-connected dc QHR value at the *V_x_* minimum). All dc guideline properties could have been determined for a good device (including those for magnetic field reversal) using the external single-series and quadruple-series configurations. (Of course the dc guidelines are a necessary, but not sufficient, condition of ac device suitability, and any future proposed ac guidelines are yet another matter.)

The ac QHR values converged to the dc QHR value under proper conditions (which were external quadruple-series connections [16], four-terminal-pair (4TP) techniques [17,18], and interchanged 1:1 ratio measurements). We wanted to demonstrate convergence to about ±1 part in 10^8^ of the dc QHR, but poor device properties limited the present uncertainty to ±1 part in 10^7^.

## 2. QHR Device, Header, and Sample Probe

The only ac QHR device available was a LEP 175 GaAs/AlGaAs heterostructure fabricated by the Laboratories d’Electronic Philips^1^ in France that we labeled ac1. K. C. Lee of NIST mounted it on one of our custom-built headers using 100 µm diameter platinum wires to avoid vibrational effects of ac currents in a magnetic field. The header, constructed from gold-patterned 1.6 mm (1/16ʺ) thick printed circuit board, had a single ground plane over most of its *back* surface to minimize wire-to-wire capacitances at the device. (This matters because Eq. (55) of Ref. [16] predicts that the largest frequency-dependent correction term in quadruply-connected ac QHR measurements is –*ω*^2^*C*_X′X′_*C*_X′X′_*R*_H_*R*_H_, which involves the squares of: the angular frequency *ω*; the summed-total *C*_X′X′_ of those wire-to-wire capacitances that have the quantum Hall voltage across them; and the quantized Hall resistance *R*_H_.) A single ground plane assures symmetry on magnetic field reversal. Tests showed that this grounded back-plane reduced wire-to-wire capacitances, with only slight increases in capacitances-to-shield, whereas a ground plane on the front surface of the header significantly increased capacitances-to-shield for similar reductions in wire-to-wire capacitance.

We predipped the eight semi-rigid coaxial cables several times in liquid helium before sample probe assembly to minimize Teflon insulation flow during cooldown. (B. W. Ricketts of the National Measurement Laboratory in Australia cautioned us that differential compression when cooling the coaxial cables squeezes the Teflon, causing it to flow during the first few cool-downs and thus stress solder joints at the coaxial socket.)

Cernox thermometers sensitive to a mK were located above and below the device, which could only be cooled to 1.5 K at maximum pumping rates (rather than the 1.3 K of our dc sample probes) because semi-rigid coaxial cables have more thermal conduction.

## 3. Device Properties

We pumped the sample probe extensively to avoid trapped gasses in the coaxial leads, and then cooled the device slowly over two days to eliminate electron-hole pair production in the device (which creates additional electrons in the two-dimensional fluid and a slow downward drift in the magnetic field positions of QHR plateaus as the pairs recombine). The device was then maintained at or below 4.2 K for 3 months.

**Fig. 1:**
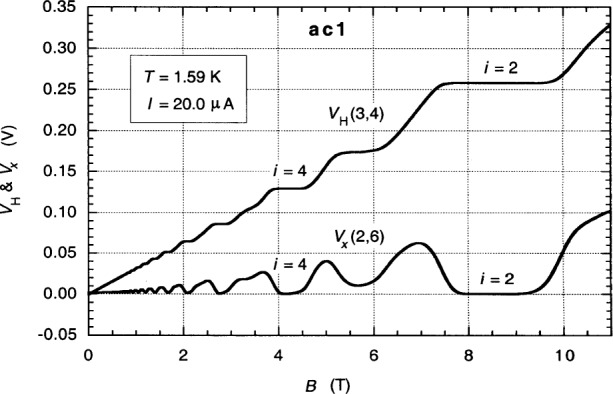
Magnetic field sweep for the central quantum Hall voltage probes *V*_H_(3,4) and the longitudinal voltage probes *V_x_*(2,6) at *I* = 20.0 µA dc and *T* = 1.59 K. The magnetic flux density *B* is in tesla. Voltage probe numbers are identified in Fig. 7.

Figure 1 shows a magnetic field sweep for the central quantum Hall voltage probes *V*_H_(3,4) and the longitudinal voltage probes *V_x_*(2,6) at a *I* = 20.0 µA dc source-drain current and *T* = 1.59 K temperature. (The device source, drain, and potential probe contacts S′, D′, 1′ – 6′ are identified in Fig. 7. Primes refer to connections at the device contact pads, while unprimed numbers and letters refer to connections outside the sample probe.)

The device looks promising in this sweep. However, three-terminal contact resistance measurements at the approximate currents that will pass through each contact in the quadruple-series mode on the *i* = 2 (12 906.4 Ω) quantum Hall plateau at 8.3 T and 1.58 K tell a different story: S′ = 9 Ω, D′ = 0.04 Ω, 1′ = 115 Ω, 2′ = 238 Ω, 3′ = 1417 Ω, 4′ = 73 Ω, 5′ = 179 Ω, and 6′ = 7644 Ω after measuring the voltages with both current directions and subtracting lead resistances. Furthermore, the 7644 Ω potential contact 6′ exhibited Corbino-like behavior: its contact resistance increased with *decreasing* current at small currents due to isolated spikes into the two-dimensional electron fluid, rather than a uniform diffusion. Current circulated around the spikes, as well as along the device. That behavior (pointed-out by K. C. Lee of NIST) would remain undetected if its contact resistance had been measured at the 20.0 µA source-drain current, rather than the ≈ 0.01 µA quadruple-series current appropriate for that probe. *It is important to measure contact resistances at the approximate multi-series connection currents, and for each current direction.* Resistances of the 1′, 3′, and 5′ potential contacts were also current-dependent, but in a more typical way: their resistances increased with increasing current.

The Corbino-like behavior of potential contact 6′ created several problems: static voltages induced when reversing dc current directions or changing ac bridge cables sometimes required minutes or hours to decay (making measurements time-consuming), and the QHR values *R*_H_ = *V*_H_/*I* often fluctuated by about 1 part in 10^7^ (1 × 10^–7^ *R*_H_) within a day or between days. A device with properties this poor would normally be immediately discarded, but no others were available. The following measurements are intended only as indicators of what could be done with a good device.

**Fig. 2:**
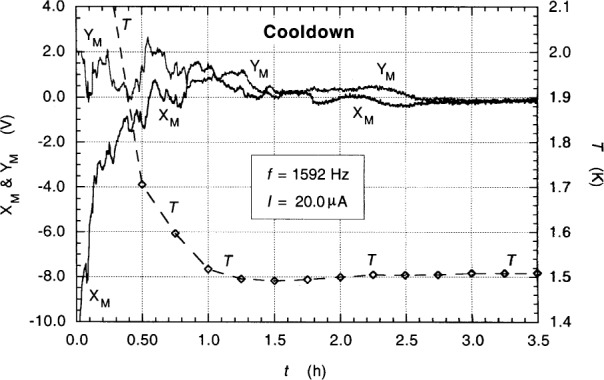
Typical cooling curves from 4.2 K each measurement day. *X*_M_ and *Y*_M_ are the in-phase and 90° out-of-phase ac QHR bridge main signal components (defined in Sec. 6). *T* is the temperature of a Cernox thermometer located just below QHR device ac1. A 6.5 V change in *X*_M_ corresponds to a 1 part in 10^6^ change in the QHR value at 1592 Hz.

The device was cooled from 4.2 K to either 1.6 K or 1.5 K in about 1 h each measurement day, but it required another 2 h to stabilize the QHR value. An example is shown in Fig. 2, where *T* is the temperature of the lower Cernox thermometer, and *X*_M_ and *Y*_M_ are the in-phase and 90° out-of-phase ac QHR bridge main signal components (defined in Sec. 6). A 6.5 V change in *X*_M_ corresponds to a 1 part in 10^6^ change in the QHR value at 1592 Hz; so the thermal stabilization problem is significant. We have no explanation for this puzzling and time-consuming feature. (It did not arise in previous temperature-dependence experiments using a dc sample probe, and was not due to thermal oscillations because the Cernox thermometers above and below the device remained synchronized and their temperatures decreased monotonically. We never filled the variable temperature insert with smaller heights of liquid helium before pumping to see if that reduced the stabilization time.)

## 4. Reference Resistors

All measurements were made in 1:1 ratio to avoid scaling ambiguities. The main reference standard was a 12 906.4 Ω wire-wound resistor labeled 12.9WW1. It was assembled from Tegam resistor components made of wire wound on mica cards and placed in a shielded container. It was designed for minimum capacitances-to-shield, trimmed to within about 6 parts in 10^6^ of the dc QHR value, and maintained in an oil bath at (25.00 ± 0.04) °C. This resistor type is used in the ac part of the NIST calculable capacitor chain from the Farad to the Ohm [19]. They are very stable, except for small linear drift rates. The 12.9WW1 drift rate is not yet determined, but typically this type drifts about 1.5 parts in 10^7^ per year.

This reference resistor was compared with the 12 906.4 Ω, *i* = 2 plateau of QHR device ac1, and also with a NL Engineering (Norman Lloyd) quadrifilar resistor (Gibbings [20] resistor) of 12 906.4 Ω nominal value that we labeled 12.9QF1. Quadrifilar resistor 12.9QF1 is maintained in a self-contained air bath and thermally-lagged with additional insulation. It lacks an independent sensor to monitor temperature stability, and looses control when the room temperature exceeds 24.5 °C. Self-heating occurs at currents above 50 µA. Its resistance value differs from the dc QHR value by about 48 parts in 10^6^, and can unpredictably change a few parts in 10^7^ over several hours, necessitating computational adjustment to a reference value (which we chose at 1592 Hz).

## 5. DC Measurements

**Fig. 3:**
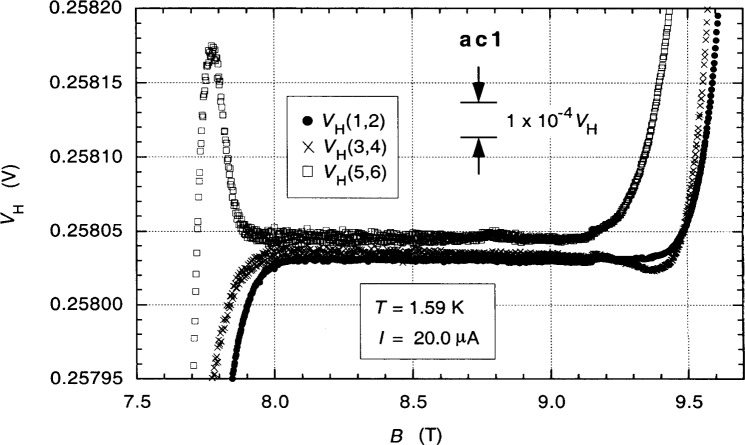
Magnetic field sweeps of the 12 906.4 Ω *i* = 2 plateaus of QHR device ac1 for the three single-series-connected quantum Hall voltage probe sets *V*_H_(1,2), *V*_H_(3,4), and *V*_H_(5,6) at 1.59 K and 20.0 µA dc.

**Fig. 4:**
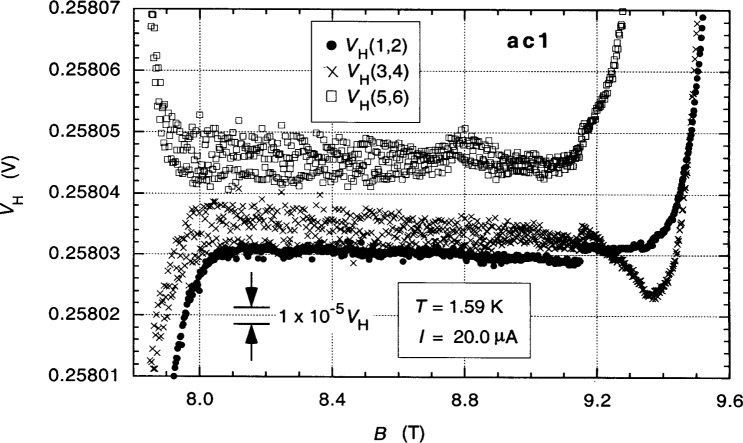
The magnetic field sweeps of Fig. 3 magnified to 4 parts in 10^6^ resolution.

It is important to understand the dc properties of the QHR device when making ac measurements; so we begin with those. Contact resistances were discussed in Sec. 3. Figure 3 shows magnetic field sweeps of the 12 906.4 Ω *i* = 2 plateaus of QHR device ac1 for the three single-series-connected quantum Hall voltage probe sets *V*_H_(1,2), *V*_H_(3,4), and *V*_H_(5,6) at 1.59 K and 20.0 µA dc using digital multimeters to measure the Hall voltages and magnetic flux density *B*. Those sweeps are magnified in Fig. 4 toto 4 parts in 10^6^ resolution. Measurements involving the 1417 Ω (probe 3) and 7644 Ω (probe 6) contacts are much noisier (with possible discrete values within the noise). Different offset voltages of the three Hall probe sets probably arise from thermoelectric effects and are of no consequence since we find later in this section that the three *V*_H_ values are comparable when including reverse-current measurements.

Large voltage shifts (about 1 part in 10^5^ of *V*_H_) appeared in all three Hall probe sets around 9.15 T in Fig. 4. They probably resulted from an hourly reading of the liquid helium level of the variable temperature insert into which the sample probe is placed since a reading occurred at that time. Liquid helium level readings were never made during subsequent measurements after daily cooldown from 4.2 K.

**Fig. 5:**
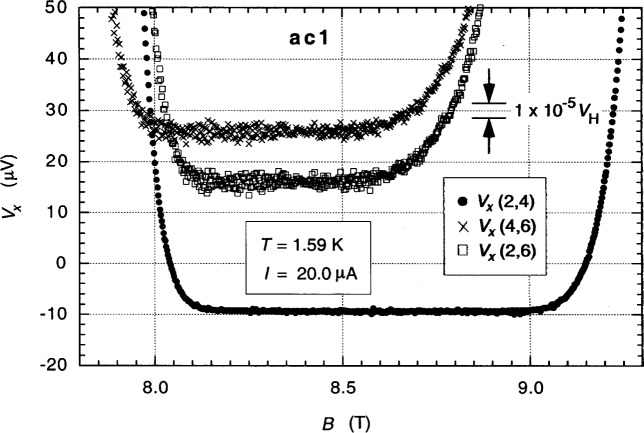
Magnetic field sweeps of the longitudinal voltage probe sets *V_x_*(2,4), *V_x_*(4,6), and *V_x_*(2,6) in the *i* = 2 plateau region at 1.59 K and 20.0 µA dc.

Figure 5 shows magnetic field sweeps of the longitudinal voltage probe sets *V_x_*(2,4), *V_x_*(4,6), and *V_x_*(2,6) in the *i* = 2 plateau region at 1.59 K and 20.0 µA dc. *V_x_* minima involving the 7644 Ω contact resistance of probe 6 were much noisier and narrower, and shifted to smaller magnetic fields. The centroid of the *V_x_*(2,6) minima is about 8.3 T.

We measured the *V_x_* minima to 1 part in 10^9^ type A, 1*σ* uncertainty at 24.0µA dc, 1.58 K, and 8.3 T with an automated potentiometeric system POTSYS [21]. Longitudinal resistance *r_x_* = *V_x_*/*I* was negligible for *r_x_* (2,4), but *r_x_* (4,6) and *r_x_* (2,6) were both 0.4 m Ω. The 7644 Ω probe 6 contact resistance clearly has an effect on *r_x_*. (*V_x_* measurements would have also been made on the high-voltage side of the device if it had been standards quality.)

The three single-series-connected *R*_H_ = *V*_H_/*I i* = 2 quantized Hall resistances *R*_H_(1,2), *R*_H_(3,4), and *R*_H_(5,6) of QHR device ac1 were compared with the 12 906.4 Ω wire-wound resistor 12.9WW1 at *I* = 29.1 µA dc, *T* = 1.58 K, and *B* = 8.3 T using an automated measurement system that reverses currents, exchanges positions of two digital multimeters, and exchanges the positions of ac1 and 12.9WW1 in the bridge. The type A, 1σ, ac1/12.9WW1 ratio values for the three single-series measurements differed from unity by (–5.97 ± 0.02) ×10^–6^, (–5.97 ± 0.02)×10^–6^, and (–5.91 ± 0.02)×10^–6^. The device therefore seems homogeneous, with the possible exception of *R*_H_(5,6) which involves the 7644 Ω contact. We note, however, that none of these three measurement sets were repeated, and we sometimes observed shifts and decays after switching. Also, we later found in the ac measurements that the *R*_H_ values were stable and reproducible only to within 1 part in 10^7^ because of the Corbino effect. Therefore we assign uncertainties of ±0.1×10^–6^ to these measurements, and assume homogeneity only within that uncertainty. (DC cryogenic current comparator measurements on a good device would allow several parts in 10^9^ uncertainties.)

**Fig. 6:**
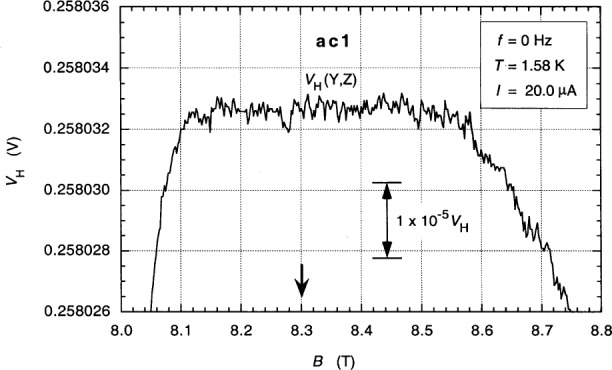
Magnetic field sweep of the quadruple-series-connected *i* = 2 plateau *V*_H_(Y,Z) at 20.0 µA dc and 1.58 K. Star locations Y and Z are defined in Fig. 7. The arrow at *B* = 8.3 T is the centroid of the *V_x_*(2,6) minimum.

**Fig. 7:**
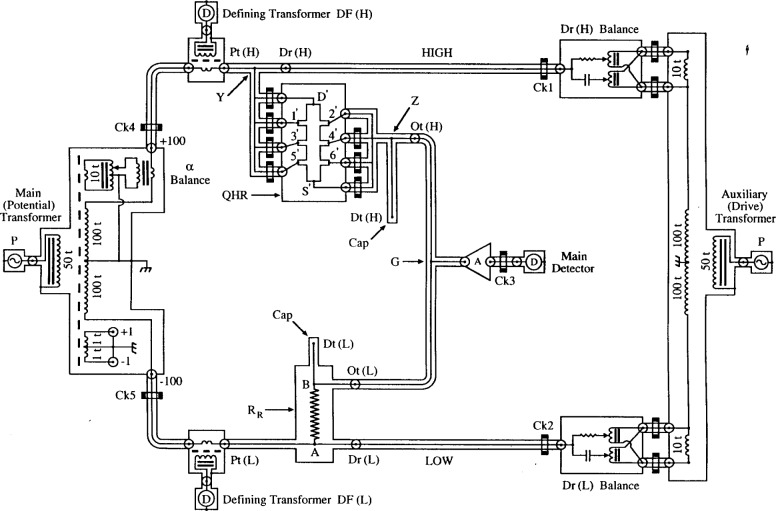
Simplified representation of the NIST multifrequency transformer ratio bridge for 1:1 ratio measurements in the three-terminal-pair (3TP) mode.

Cage, Jeffery, and Matthews [16] predicted that devices with two sets of external quadruple-series connections allow dc guideline properties and dc and ac QHR values with small and predicable quadratic frequency dependences to be measured with all sample probe leads attached at the device. Figure 6 is a magnetic field sweep of the quadruple-series-connected *i* = 2 plateau *V*_H_(Y,Z) at 20.0 µA dc and 1.58 K, where room temperature locations Y and Z are defined in Fig. 7. The plateau appears flat in this 1 part in 10^6^ resolution plot. However, the 1 part in 10^8^ resolution plot of Fig. 12 that will be shown for ac currents suggests the dc “plateau” is likely an inverted “U”, with no flat region. Furthermore, Fig. 12 looks like Fig. 6 when plotted at 1 part in 10^6^ resolution. DC cryogenic current comparator (CCC) measurements would have the resolution to confirm this inverted “U” supposition, but it was not worth moving CCC apparatus between laboratories for this poor-quality device. (CCC measurements would have been made across the plateau regions of *V*_H_(Y,Z), *V*_H_(1,2), *V*_H_(3,4), and *V*H(5,6) to several parts in 10^9^ resolution on a good device.)

We see from Eq. (55) of Ref. [16] that *V*_H_(Y,Z) is primarily the quantity *V*_H_(3,4) – *V_x_*(2,6) in homogeneous, quadruple-series-connected devices. That is of no consequence in good devices when cooled to temperatures where *V_x_*(2,6) is negligible over the *V_x_* minima region. But *V_x_*(2,6) is not negligible here because of poor contacts. That may be the source of the possible inverted “U” shape of *V*_H_(Y,Z).

The quadruple-series-connected resistance *R*_H_(Y,Z) was compared with the 12 906.4 Ω wire-wound reference resistor 12.9WW1 at *I* = 29.1 µA dc, *T* = 1.58 K, and *B* = 8.3 T using the automated double-multimeter measurement system. The ratio differed from unity by (–6.03 ± 0.02) × 10^–6^. That value is slightly smaller than the three single-series ratios because the quadruple-series mode measures *R*_H_–[*r_x_*(2,4) + *r_x_*(4,6)] [16] and the value of *r_x_*(4,6) is 3 × 10^–8^ *R*_H_. The dc measurements would have been repeated at least once, and at different places along the plateau, but programmatic constraints interceded. As mentioned earlier, we found during subsequent ac measurements for this device that the QHR values were stable and reproducible only to within ±1 part in 10^7^ because of the Corbino effect. Therefore we assign a dc value of [1–(6.0 ± 0.1) ×10^–6^] to this 1:1 ratio. No effort was made to see how well the *i* = 2 dc quadruple-series QHR value of this poor-quality device approximates the 12 906.403 5 Ω von Klitzing constant *R*_K_ since we were only investigating convergence of ac and dc values in this experiment, and would in any case not use ac1 as a standard.

## 6. AC Bridge

The NIST multifrequency transformer bridge can measure 1:1, 2:1, and 10:1 ratios. We used interchanged 1:1 ratios here to minimize ambiguities, and the same set of bridge windings in every interchanged measurement. Figure 7 shows a simplified representation of the three-terminal-pair (3TP) mode in 1:1 ratio. (“Terminal-pair” is an accessible coaxial connection (port) consisting of an inner conductor and its shield. “Three” is the number of terminal-pair connections of a 4-port standard that meet the 4TP balance conditions [18].)

A 20.0 µA rms drive current, generated by primary voltage source signal P and an auxiliary (drive) transformer, passes through: coaxial cable on the High (H) side of the bridge to drive port Dr(H) at external “star” connector Y; QHR device ac1 (connected in quadruple-series between external stars Y and Z; coaxial cables between out ports Ot(H) and Ot(L) on the High and Low (L) sides of the bridge; reference resistor 12.9WW1 (shown here as resistor *R*_R_, with internal connection points A and B) to drive port Dr(L); and back to the auxiliary transformer. Passive coaxial chokes [22] (current equalizers) Ck1 and Ck2 (with 20 turns wound around a magnetic core) assure nearly equal and opposite currents in the inner and outer conductors of the coaxial drive cables.

The primary voltage signal P is also supplied to the main (potential) transformer. Main balance is achieved by adjusting the six-decade in-phase α dials and 90° out-of-phase β dials on the main (potential) transformer until the in-phase and out-of-phase impedance signal components *X*_M_ and *Y*_M_ are amplified at A and nulled in the main lock-in detector D. There is negligible current in the inner conductor to the main detector at balance. Coaxial choke Ck3 then assures negligible current in the outer conductor. The inner conductor of “star” connector G is at virtual ground at balance. (The β adjustment network is not shown in the figure. It consists of a six-decade β balance identical to the α balance, a 10 nF mica capacitor in a 25 °C oil bath, and a choked coaxial cable also inserted into star G.)

Defining transformers DF(H) and DF(L), and lock-in detectors D assure negligible current in the inner conductors at the High and Low potential ports Pt(H) and Pt(L) by adjusting four-decade in-phase and out-of-phase inductive voltage dividers (IVDs) Dr(H) Balance and Dr(L) Balance in the drive circuit to null the in-phase and out-of-phase defining transformer detector signals DF(H) and DF(L). Coaxial chokes Ck4 and Ck5 in the potential circuit approximate the zero current condition in the outer conductors at potential ports Pt(H) and Pt(L). Brass caps cover the outer conductors of the open detection ports Dt(H) and Dt(L) to minimize noise.

Systematic errors may arise if the network is over-choked or under-choked. No effort has yet been made to vary the number or location of chokes. We chose the coaxial cable between contact 2′ and star connector Z to be unchoked to avoid over-choking the QHR “standard” itself because that cable is near ground potential and carries the smallest current. The outer shields of all bridge components are grounded via one point at the main (potential) transformer. (The effect of adding extra ground connections will be tested in the future.)

This circuit satisfies the 3TP conditions at *both* the QHR “standard” and the reference resistor: (1) negligible current at the inner and outer conductors of potential ports Pt(H) and Pt(L), and therefore no in-phase currents in the potential cables to external connection star Y of the QHR “standard” or to internal connection point A of the reference resistor; (2) negligible current and voltage in the inner and outer conductors to the main detector D; (3) the same current to/from the QHR “standard” and the reference resistor at connection star G (where the inner conductor is at virtual ground at balance); and (4) equal and opposite currents in the inner and outer conductors of cables between external connection star Z of the QHR “standard” and internal connection point B of the resistor. (In addition, detection ports Dt(H) and Dt(L) have no current since they are open-circuited. That meets part of the 4TP definition. However, ports Dt(H) and Dt(L) are not at ground potential because of cable loses to star G.)

**Fig. 8:**
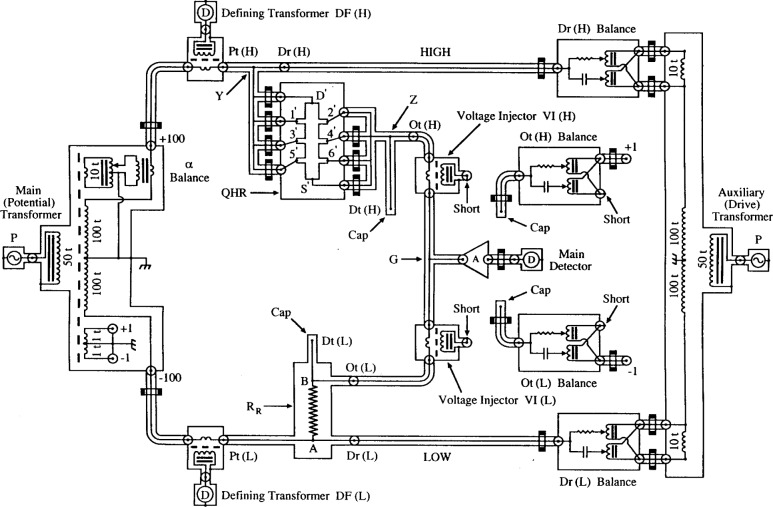
Modifications of the 3TP circuit in Fig. 7 to minimize cabling changes between 3TP and 4TP measurement modes.

The 3TP circuit of Fig. 7 works perfectly well, but Fig. 8 shows modifications to minimize cabling changes between the 3TP and 4TP measurement modes. Voltage injectors VI(H) and VI(L) were added to the coaxial cables leaving the out ports Ot(H) and Ot(L), and the VI output windings were shorted. This only increased cable impedances from each out port Ot(H) or Ot(L) to star G by 0.5 mΩ, 4.4 pF, and 1 µH. (The purposes of IVDs Ot(H) Balance and Ot(L) Balance and their capped cables will be explained for the 4TP mode. They have no influence on 3TP measurements.)

3TP balances are made in an iterative process using amplifier A, lock-in detector D, and the bridge main adjustment dials α and β to null the *X*_M_ and *Y*_M_ impedance signals at star G. A second lock-in detector D moves between defining transformers DF(H) and DF(L) to assure negligible currents in the inner conductors at potential ports Pt(H) and Pt(L) by adjusting IVDs Dr(H) Balance and Dr(L) Balance. This second detector is always replaced with a shorted inner/outer connector at the DF(H) or DF(L) output winding, and is completely removed on final bridge balance.

We initially inserted a “combining network” like that of Fig. 6 in Cutkosky’s paper [18] between ports Ot(H), Dt(H) and ports Ot(L), Dt(L) to approximate the 4TP definition. (An example is shown in Fig. 3 of Ref. [23] for an earlier NIST bridge.) However, measurement errors arose that were sometimes as large as 18 parts in 10^6^ under “proper” 4TP balance conditions using different internal resistance values of the combining network when measuring 10:1 resistance ratios. (Perhaps this resulted from using out-of-phase β signal injection into a ground potential location of the combining network, rather than into a location on the higher potential side. This will be checked in future.)

**Fig. 9:**
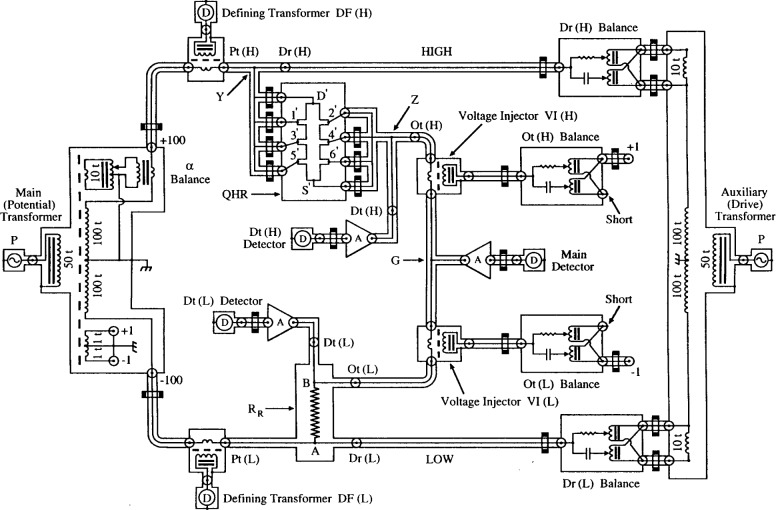
NIST multifrequency bridge circuit representation for 1:1 ratio measurements in the 4TP mode.

We therefore removed the combining network and made all 4TP measurements by “brute force” using five in-phase and five out-of-phase balances, as indicated in Fig. 9. Injectors VI(H) and VI(L) insert voltages into the coaxial cables between out ports Ot(H) and Ot(L) to make up for cable loses such that the in-phase and out-of-phase signals are nulled at detection ports Dt(H) and Dt(L), as well as at star G. This is accomplished with IVD adjustments Ot(H) Balance and Ot(L) Balance to null the in-phase and out-of-phase signals at ports Dt(H) and Dt(L) using amplifiers A and lock-in detectors D. Thus the inner conductors of ports Dt(H), Dt(L), and G are all at virtual ground at balance. Those two IVDs are connected to +1 turn and –1 turn taps on a separate winding of the main (potential) transformer. (It is possible that they slightly load the potential transformer. That will be tested in future by connecting them to existing +1 and –1 or +10 and –10 taps (not shown) of the drive transformer.)

Cable lengths were carefully matched to minimize errors. Inner and outer contacts of every British Post Office (BPO) cable connector were individually tested and polished to avoid ratio measurement shifts of order 1 part in 10^7^. (All outer contacts of a recent BPO purchase were too loose, and the inner contacts very tight; so the connections “felt” good, but occasionally caused large shifts.) Each week we twisted every connector several times to maintain polish and avoid shifts of order 1 part in 10^8^. Leakage resistances of all BPO connectors, the few BNC connectors, and all coaxial cables are greater than 10^14^ Ω. They were maintained to that value with dust covers on all open connectors.

4TP balances use the 3TP procedures listed above to obtain a preliminary main balance and null currents at potential ports Pt(H) and Pt(L). Amplifier A and lock-in detector D, used for nulling the *X*_M_ and *Y*_M_ signals at main balance star G, is then sequentially moved to detector ports Dt(H) and Dt(L), and their in-phase and out-of-phase signals nulled by adjusting IVDs Ot(H) Balance and Ot(L) Balance. (Brass caps cover any open outer conductors at ports Dt(H), Dt(L), and star G to minimize noise.)

It requires several iterations and some experience to simultaneously null all three sets of low voltage in-phase and out-of-phase signals because the main, Ot(H), and Ot(L) balance adjustments interact. Although tedious to initially determine at each frequency, the bridge adjustment parameters are reproducible, and should provide a close approximation to ideal 4TP measurements.

The 4TP bridge, at balance, measures the ratio of the QHR “standard” (with its in-phase impedance component defined between external connection stars Y and Z) and the wire-wound resistor (defined between internal connection points A and B). (Figure 4 of the Cage, Jeffery, and Matthews equivalent circuit model [16] defines the QHR “standard” at access ports Dr(H), Pt(H), Dt(H), and Ot(H). Dr(H), Dt(H), and Ot(H) are located on stars Y and Z. Pt(H) is separated from star Y by a small length of coaxial cable. The additional impedances from stars Y and Z to those four access ports have negligible effect on the in-phase component of the ac QHR value in our bridge at 4TP balance.)

**Fig. 10:**
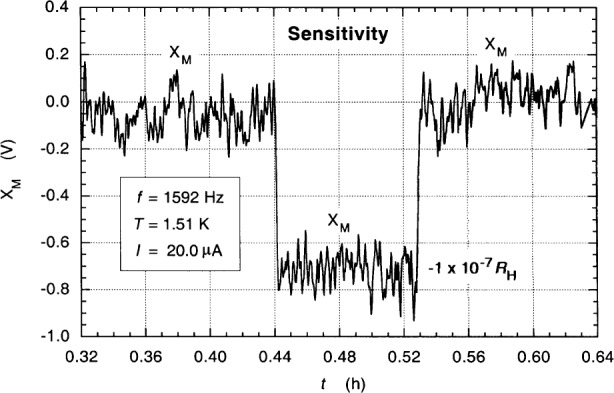
NIST multifrequency bridge sensitivity of the main in-phase impedance signal component *X*_M_ for the ac1/12.9WW1 ratio with a –1 part in 10^7^ change of the *α* dials setting.

Figure 10 demonstrates the bridge sensitivity of the balanced and amplified in-phase main signal component *X*_M_ for the ac1/12.9WW1 ratio with a –1 part in 10^7^ change of the dial settings. The bridge has a 5 parts in 10^9^ resolution at 1592 Hz and 20.0 µA. This is comparable with any bridge in the NIST calculable capacitor chain from the Farad to the Ohm [19]. Its in-phase resolution increases at lower frequencies and decreases at higher frequencies.

## 7. AC QHR Measurements

**Fig. 11:**
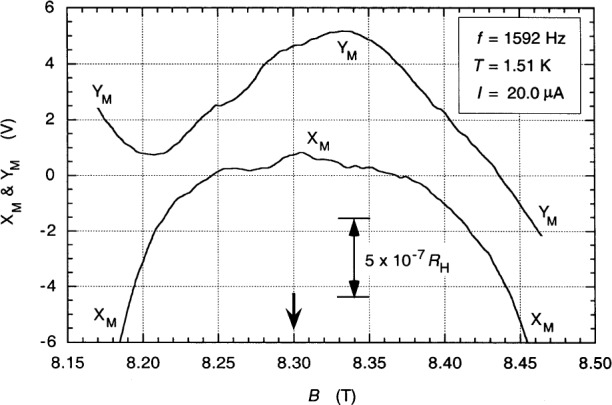
Magnetic field sweep over the quadruple-series-connected *i* = 2 plateau *V*_H_(Y,Z) at 1.51 K compared in 4TP mode with wire-wound resistor 12.9WW1 at 20.0 µA rms and 1592 Hz. The 90° out-of-phase signal component *Y*_M_ is offset for clarity from the in-phase main lock-in detector signal component *X*_M_. The arrow at *B* = 8.3 T is the centroid of the *V_x_*(2,6) minimum.

**Fig. 12:**
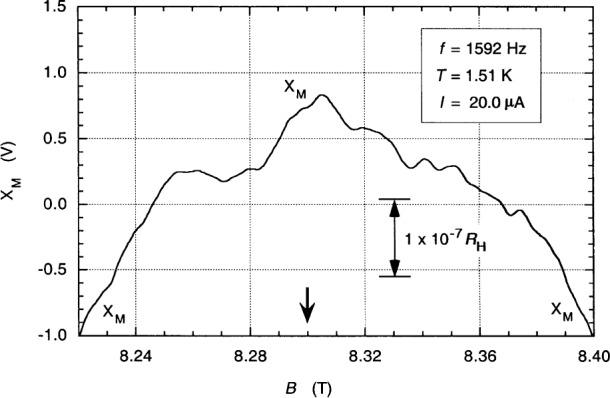
Magnified version of the Fig. 11 *X*_M_ signal.

Figure 11 shows a magnetic field sweep over the quadruple-series-connected *i* = 2 plateau *V*_H_(Y,Z) at 1.51 K compared in 4TP mode with the 12.9 kΩ wire-wound resistor 12.9WW1 at 20.0 µA rms and 1592 Hz. The 90° out-of-phase signal component *Y*_M_ is offset for clarity from the in-phase main lock-in detector signal output *X*_M_. *X*_M_ and *Y*_M_ sweeps at 700 Hz, 3000 Hz, and 5000 Hz have similar shapes and magnetic field locations. Figure 12 shows the Fig. 11 *X*_M_ signal magnified. The ac QHR “plateau” is an inverted “U” with no flat region. (As mentioned before in Sec. 5, it appears just as flat as the dc quadruple-series plateau of Fig. 6 when plotted to that resolution.) The “peak” feature near 8.3 T was reproducible, and occurs at the same magnetic flux density as the *V_x_*(2,6) minimum. We suspect it is due to the [*V*_H_ – *V_x_*(2,6)] aspect of quadruple-series measurements, and would not appear on plateaus of good devices when cooled enough that *V_x_*(2,6) was negligible. This feature may contribute to the ±1 part in 10^7^ fluctuations in the resistance ratio. AC bridge measurements were always made at the *B* = 8.3 T value used for dc measurements.

**Fig. 13:**
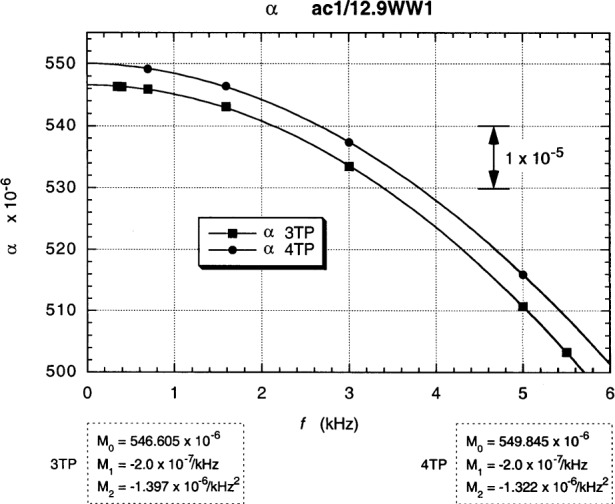
3TP and 4TP plots of main bridge dial “raw data” readings *α* vs frequency *f* at 20.0 µA rms, 8.3 T, and 1.51 K when the device and reference resistor are in their “normal” positions, with ac1 on the High side of the bridge and 12.9WW1 on the Low side. A perfect unity ratio, with no frequency dependences or bridge corrections, would be *α* = 555.555. A change of +1.0 in the *α* dials reading represents a +1.0 part in 10^6^ shift in the in-phase resistance ratio signal *X*_M_.

3TP and 4TP plots of *α* vs *f* in Fig. 13 at 20.0 µA rms, 8.3 T, and 1.51 K are the in-phase six-decade main dial readings of the resistance ratio ac1/12.9WW1 vs frequency when the device and reference resistor are in their “normal” positions, with ac1 on the High side of the bridge and 12.9WW1 on the Low side. The bridge is constructed such that: a perfect unity ratio, with no frequency dependences or bridge corrections, would be *α* = 555.555, where a change of +1.0 in the *α* dials reading represents a +1.0 part in 10^6^ shift in the in-phase ratio signal *X*_M_. (We deliberately plot this “raw data” in that format to indicate that *no* bridge or cable corrections are applied at this stage.) 3TP balances were achieved between 350 Hz and 5500 Hz; 4TP balances between 700 Hz and 5000 Hz. (A 5500 Hz 4TP datum point was not taken when it appeared for a while that the project was going to be terminated.)

Corbino-like behavior of the 7644 Ω potential contact meant that static voltages induced when changing bridge leads sometimes required minutes, or even hours, to decay. As a result, every measurement has a ±1 part in 10^7^ uncertainty; which is frustrating when compared with the 5 parts in 10^9^ resolution.

Second-order polynomial fits to the data in Fig. 13 assume a constant term *M*_0_, a linear frequency term *M*_1_*f*, and a quadratic frequency term *M*_2_*f*^2^. Coefficients *M*_0_, *M*_1_, and *M*_2_ are listed in the figure. Large linear and quadratic frequency dependences occur for both 3TP and 4TP measurements.

**Fig. 14:**
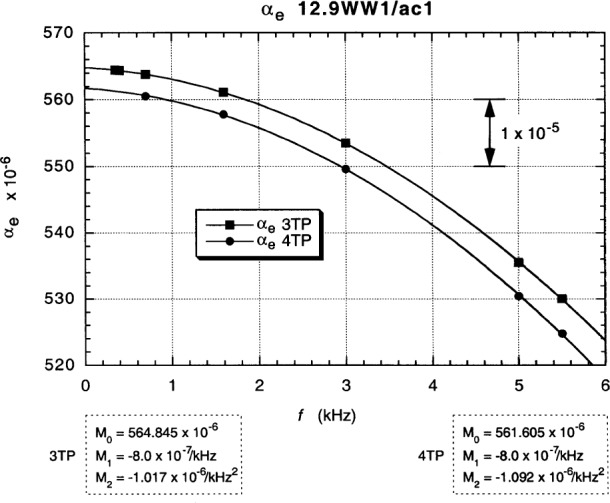
Main dial 3TP and 4TP “raw data” readings *α*_e_ vs *f* with the device and reference resistor positions “exchanged” in 1:1 ratio measurements: 12.9WW1 on the High side of the bridge and ac1 on the Low side.

Figure 14 shows the 3TP and 4TP main dial “raw data” readings *α*_e_ vs *f* with the device and reference resistor positions “exchanged” in the 1:1 ratio measurements: 12.9WW1 on the High side of the bridge and ac1 on the Low side. We tried two exchange methods: (1) physically moving cables between the Dr(H), Pt(H), Ot(H) ports and the Dr(L), Pt(L), Ot(L) ports; and (2) exchanging the other end of those coaxial cables at the High and Low taps of the main (potential) and auxiliary (drive) transformers. Both methods gave similar results. The later exchange method was used because it is physically easier and involves less change in relative cable positions. There is a large (6.0 ± 0.1) × 10^–7^/kHz difference in linear frequency dependence between the “exchanged” and “normal” position fits of Figs. 13 and 14.

The in-phase *ac bridge* contributions *α*_0_ to the *α* and *α*_e_ measurements of Figs. 13 and 14 are obtained by averaging the sum of the “normal” and “exchanged” “raw data” at each frequency to obtain interchanged bridge in-phase components *α*_0_:

$$\alpha_{0}=\left(\alpha +\alpha_{e}\right)/2.$$ (1)

**Fig. 15:**
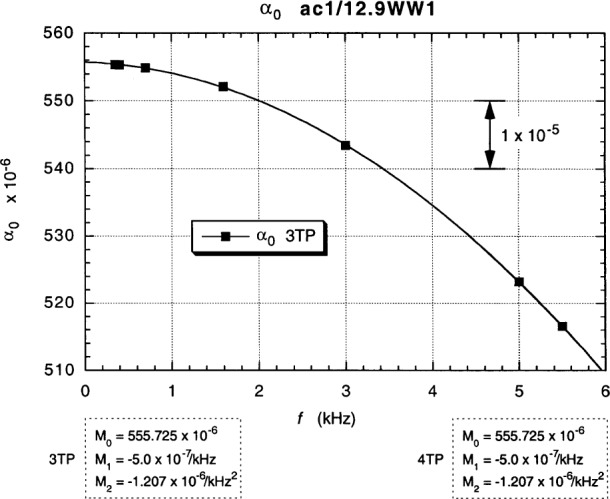
In-phase ac bridge contributions *α*_0_ to the *α* and *α*_e_ measurements of Figs. 13 and 14, obtained by averaging the sums of the “normal” and “exchanged” “raw data”. These *α*_0_ vs *f* results are the same for both 3TP and 4TP measurements.

Figure 15 plots these *α*_0_ vs *f* results, which are the same for both 3TP and 4TP measurements. There is a significant offset from the ideal unity value: (555.725 – 555.555) × 10^–6^ = (+1.7 ± 1.0) × 10^–7^. Note also the large (–5.0 ± 0.1) × 10^–7^/kHz linear frequency dependence. A linear dependence term was unexpected, but definitely exists given the ±1 part in 10^7^ data uncertainties. The (–1.207 ± 0.010) × 10^–6^/kHz^2^ quadratic dependence is very large.

**Fig. 16:**
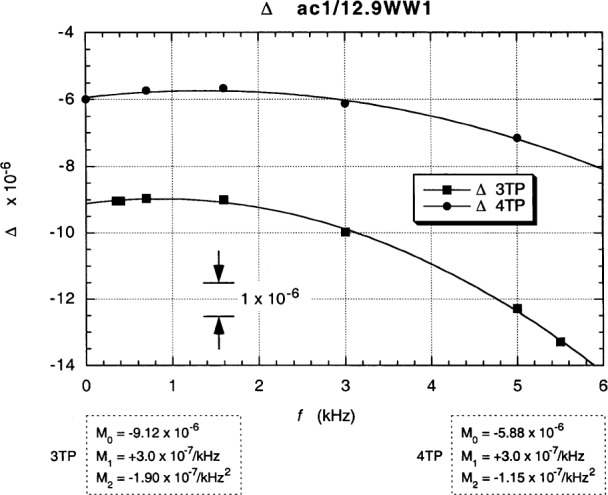
Interchanged in-phase *∆* deviations from the unity resistance ratio ac1/12.9WW1, obtained by averaging the differences between the “normal” and “exchanged” “raw data” of Figs. 13 and 14. The 4TP interchanged ac measurements converge within the ±1 part in 10^7^ uncertainty to the –6.0 parts in 10^6^ dc value.

Interchanged in-phase deviations *∆* from the unity *resistance ratio* are obtained by averaging the difference between the “normal” and “exchanged” “raw data” for each frequency:

$$\Delta =\left(\alpha -\alpha_{e}\right)/2$$ (2)

as shown in Fig. 16. Note that the 4TP interchanged ac QHR deviation measurements converge within the ±1 part in 10^7^ relative standard deviation uncertainty to the dc value of (–6.0 ± 0.1) parts in 10^6^, whereas the interchanged 3TP measurements are in error by (–3.2 ± 0.1) parts in 10^6^ on extrapolation to dc when using the “raw data” with no cable corrections. Cable corrections are not necessary in our 4TP bridge of Fig. 9 because voltage injectors VI(H) and VI(L) make up losses in the out cables, and Dt(H) and Dt(L) are both at zero voltage. (However, the ac QHR “standard” is defined between external stars Y and Z; it therefore includes effects due to coaxial cables from the QHR device to those stars, as shown in the equivalent circuit model of Ref. [16].)

Differences in cable impedance between star Z to star G and point B to star G contribute to the –3.2 parts in 10^6^ error in the 3TP mode from 4TP at dc. Only –2.4 of this –3.2 parts in 10^6^ error is due to differences in the out cable resistances. Furthermore, it would be difficult to correct for these differences to high accuracy: a 0.13 mΩ variation of a connector contact resistance in the out cables would change the measured resistance ratio by 1 part in 10^8^. 3TP measurements are not adequate.

Note that the bridge linear frequency dependence is *larger* in magnitude than that of the resistance ratio: (–5.0 ± 0.1) × 10^–7^/kHz compared with (+3.0 ± 0.1) × 10^–7^/kHz, and that the bridge quadratic frequency dependence is *ten times larger*: (–12.07 ± 0.10) × 10^7^/kHz^2^ compared with (–1.15 ± 0.10) × 10^–7^/kHz^2^. Bridge effects can dominate ac QHR *α* or *α*_e_ frequency-dependence measurements, and are unique to each bridge. It is thus crucial to perform *interchanged* measurements to obtain correct frequency dependences of resistance ratios.

Fits to the data in Figs. 13 – 16 are excellent, and are *self-consistent* for all three terms of every polynomial:

$$2{\left(M_{0}\right)}_{\alpha_{0}}={\left(M_{0}\right)}_{\alpha }+{\left(M_{0}\right)}_{\alpha_{e}}\;\text{and}\;2{\left(M_{0}\right)}_{\Delta }={\left(M_{0}\right)}_{\alpha }-{\left(M_{0}\right)}_{\alpha_{e}}$$ (3a)

$$2{\left(M_{1}\right)}_{\alpha_{0}}={\left(M_{1}\right)}_{\alpha }+{\left(M_{1}\right)}_{\alpha_{e}}\;\text{and}\;2{\left(M_{1}\right)}_{\Delta }={\left(M_{1}\right)}_{\alpha }-{\left(M_{1}\right)}_{\alpha_{e}}$$ (3b)

$$2{\left(M_{2}\right)}_{\alpha_{0}}={\left(M_{2}\right)}_{\alpha }+{\left(M_{2}\right)}_{\alpha_{e}}\;\text{and}\;2{\left(M_{2}\right)}_{\Delta }={\left(M_{2}\right)}_{\alpha }-{\left(M_{2}\right)}_{\alpha_{e}}$$ (3c)

The measurement uncertainties are ±1 part in 10^7^. Unfortunately the device characteristics prevented measurements to parts in 10^8^. That would have rigorously tested the quality of fits and convergence to the dc value.

## 8. AC Quadrifilar Resistor Measurements

**Fig. 17:**
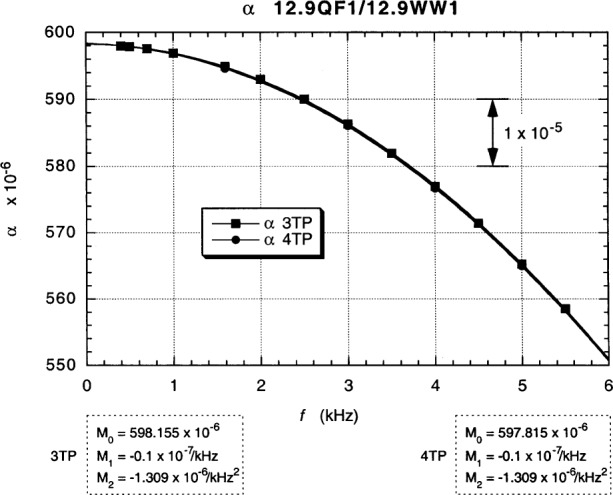
“Raw data” 3TP and 4TP plots of *α* vs *f* at 20.0 µA rms, 8.3 T, and 1.51 K when 12.9QF1 and 12.9WW1 are in their “normal” bridge positions.

**Fig. 18:**
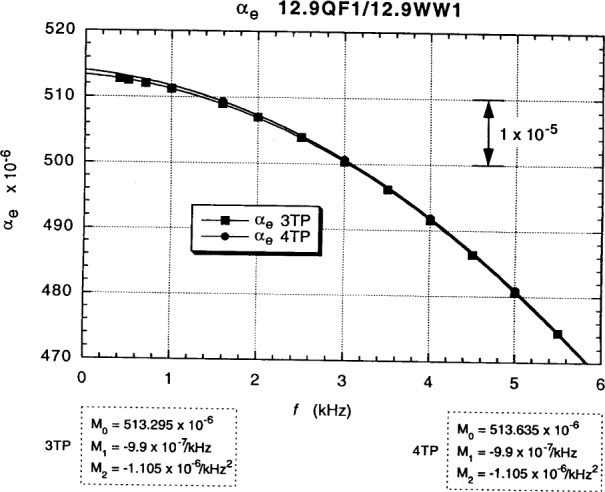
“Raw data” 3TP and 4TP plots of *α* vs *f* with 12.9QF1 and 12.9WW1 “exchanged” in the bridge.

**Fig. 19:**
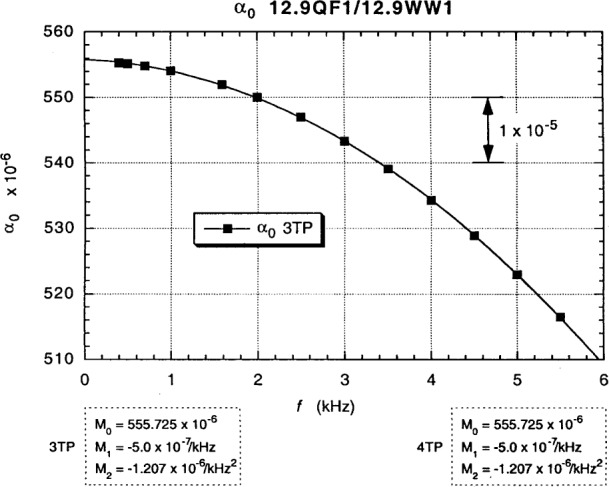
AC bridge contributions *α*_0_ to the *α* and *α*_e_ measurements, obtained by averaging the sums of “normal” and “exchanged” “raw data” of Figs. 17 and 18. These *α*_0_ vs *f* results are the same for both 3TP and 4TP modes, and are identical to those of Fig. 15 for interchanged ac1/12.9WW1 measurements.

**Fig. 20:**
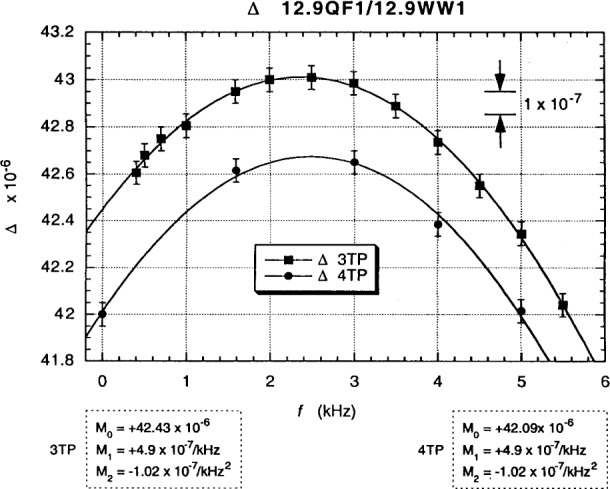
Interchanged in-phase deviations *∆* from the unity resistance ratio 12.9QF1/12.9WW1, obtained by averaging differences between the “normal” and “exchanged” “raw data” of Figs. 17 and 18. The 4TP interchanged measurements converge within the ±1 part in 10^7^ uncertainty to the +42.0 parts in 10^6^ dc value.

Similar 20.0 µA rms ac measurements and 29.1 µA dc measurements comparing the 12 906.4 Ω quadrifilar resistor 12.9QF1 with the wire-wound reference resistor 12.9WW1 had been made nine months earlier. They are shown in Figs. 17–20. The ratio value was remeasured each day at 1592 Hz and adjusted to account for shifts of resistor 12.9QF1. Each datum point has a ±5 parts in 10^8^ 1*σ* type A uncertainty. That could be reduced with more frequent measurements of the 12.9QF1 resistance shift, but this experiment was only intended as an initial mapping of bridge parameters.

“Raw data” 4TP interchanged resistance ratio deviation measurements *∆* again converge to the (42.0 ± 0.1) parts in 10^6^ dc value in Fig. 20. The “raw data” 3TP convergence error is about 4 parts in 10^7^. That is much smaller than the –3.2 parts in 10^6^ error in Fig. 16 for the ac1/12.9WW1 ratio because out cable lengths (points B to star G) between the two resistors are more closely-matched than the star Z to star G length to the ac QHR “standard”.

All three bridge *α*_0_ polynomial terms of Fig. 19 are *identical* to those in Fig. 15 for both 3TP and 4TP measurements. Thus the bridge is very stable, and provides consistent results whether comparing the wire-wound resistor with a QHR “standard” or with a quadrifilar resistor. Linear frequency dependence of the 12.9QF1/12.9WW1 ratio is larger than that of the ac1/WW1 ratio: (+4.9 ± 0.1) × 10^–7^/kHz compared with (+3.0 ± 0.1) × 10–7/kHz). The quadratic dependence is similar: (–1.02 ± 0.10) × 10–7/kHz2 compared with (–1.15 ± 0.10) × 10–7/kHz2.

Inadequate room airconditioning prevented direct comparisons of device ac1 with quadrifilar resistor 12.9QF1. Room temperatures rose above 24.5 °C each day the magnet was used, causing 12.9QF1 to lose temperature control. Thus ac1/12.9QF1 intercom-parisons await the move to a new laboratory. However, the ac1/12.9WW1 and 12.9QF1/12.9WW1 intercom-parisons imply that

$$\begin{array}{l} \text{ac}1/12.9\text{QF}1=\left[1-\left(48.00\pm 0.14\right)\times 10^{-6}\right]\\ -\left[\left(1.90\pm 0.14\right)\times 10^{-7}/\text{kHz}\right]f\\ -\left[\left(1.3\pm 1.4\times 10^{-8}\right)/{\text{kHz}}^{2}\right]f^{2}. \end{array}$$ (4)

## 9. Frequency Dependences

Quadratic frequency dependences will be discussed in a future paper [24], but we see here that the bridge quadratic dependence was at least ten times larger than for any resistance ratio. Large linear frequency dependences appeared not only in measurements involving the ac QHR “standard,” but also in the ac bridge, and in comparisons of two different types of resistors. Because of programmatic constraints, and since the device was of such poor quality, we did not pursue sources of the linear frequency dependence of the bridge, the ac1/12.9WW1 resistance ratio, or the 12.9QF1/12.9WW1 ratio. (Such as: moving the unchoked cable from probe 2 to other probe positions; adding chokes to the network; removing chokes; and verifying that the currents are indeed equal and opposite in the inner and outer conductors of every choke to test Schurr and Melcher's observations [25] that linear frequency dependences can be induced by improper choking and imperfect current equalization.)

## 10. Conclusions

DC QHR guideline properties and the dc and ac QHR values can be determined during one cooldown using single-series and quadruple-series connections outside the sample probe. The ac QHR values converged to the dc QHR value to within ±1 part in 10^7^ with a poor device when using external quadruple-series connections and four-terminal-pair measurements. Convergence could be tested in the future to an order of magnitude smaller uncertainty with good devices. It was crucial to use 1:1 resistance ratios and make interchanged measurements to remove dominant ac bridge frequency-dependence effects.

## Footnotes

1 Certain commercial equipment, instruments, or materials are identified in this paper to foster understanding. Such identification does not imply recommendation or endorsement by the National Institute of Standards and Technology, nor does it imply that the materials or equipment identified are necessarily the best available for this purpose.
